# Patients' perspectives on taking warfarin: qualitative study in family practice

**DOI:** 10.1186/1471-2296-5-15

**Published:** 2004-07-21

**Authors:** Guilherme Coelho Dantas, Barbara V Thompson, Judith A Manson, C Shawn Tracy, Ross EG Upshur

**Affiliations:** 1 Primary Care Research Unit, Sunnybrook and Women's College Health Sciences Centre, 2075 Bayview Avenue, Room E3-49, Toronto, ON M4N 3M5 Canada; 2 Department of Family and Community Medicine, Sunnybrook and Women's College Health Sciences Centre, 2075 Bayview Avenue, Toronto, ON M4N 3M5 Canada; 3 Department of Public Health Sciences, University of Toronto, McMurrich Building, 12 Queen's Park Crescent W., Toronto, ON M5S 1A8 Canada

**Keywords:** Anticoagulation, Family Practice, Patient Preference, Primary Care, Qualitative Research

## Abstract

**Background:**

Despite the well-documented benefits of using warfarin to prevent stroke, physicians remain reluctant to initiate therapy, and especially so with the elderly owing to the higher risk of hemorrhage. Prior research suggests that patients are more accepting of the risk of bleeding than are physicians, although there have been few qualitative studies. The aim of this study was to employ qualitative methods to investigate the experience and perspective of individuals taking warfarin.

**Methods:**

We conducted face-to-face interviews with 21 older patients (12 male, 9 female) who had been taking warfarin for a minimum of six months. Participants were patients at a family practice clinic situated in a large, tertiary care teaching hospital. We used a semistructured interview guide with four main thematic areas: decision-making, knowledge/education, impact, and satisfaction. Data were analysed according to the principles of content analysis.

**Results and Discussion:**

Participants tended to have minimal input into the decision to initiate warfarin therapy, instead relying in great part on physicians' expertise. There appeared to be low retention of information received regarding the therapy; half the patients in our sample possessed only a superficial level of understanding of the risks and benefits. This notwithstanding, participants reported a high level of satisfaction with the care provided and a low level of impact on their day-to-day lives.

**Conclusions:**

Minimal patient involvement in the initial decision and modest knowledge did not appear to diminish satisfaction with warfarin management. At the same time, care providers exert a tremendous influence on the initiation of warfarin therapy and should strive to incorporate patient preferences and expectations into the decision-making process.

## Background

Warfarin therapy is an effective anticoagulant indicated for the prophylaxis and/or treatment of venous thrombosis and atrial fibrillation (AF), the most common cardiac arrhythmia in older individuals [1]. Oral anticoagulation with warfarin is known to reduce the risk of disabling stroke; indeed, the benefits of oral anticoagulation have been demonstrated in a number of systematic reviews, providing high evidential support for prophylaxis [2,3]. Published guidelines on the management of AF emphasize the importance of warfarin therapy for the prevention of stroke [4]. Likewise, there is convincing evidence that long-term warfarin therapy is a highly effective method of preventing recurrent venous thromboembolism [5].

Despite the strong evidence base and the endorsement of warfarin therapy by authoritative guidelines, current prescribing patterns of warfarin remain something of a puzzle. Bungard and associates have described the 'real world' use of warfarin therapy as "sub-optimal" [6] and have estimated that only 15% to 44% of patients eligible for anticoagulation are actually prescribed warfarin [7]. Hart concurs that the use of warfarin is poor, noting that "it is often given to patients who benefit minimally, while those patients who would benefit most are not treated" [8].

Bungard and his colleagues recently conducted a systematic review of the reasons for the underuse of warfarin, identifying patient, provider, and system factors as well as identifying limitations in research studies and arguing for further research into these factors [6]. Studies employing trade-off methods to determine the risk/benefits threshold where therapy becomes acceptable have demonstrated that patients are more willing to assume risk when better informed about the medication [9]. Physicians, however, are reluctant to prescribe warfarin to elderly patients owing to concerns regarding compliance, a perceived risk of falls, and the lack of randomized controlled trial evidence in this patient population [7]. A recent study reporting the findings of interviews with individuals with a history of AF indicated that patients' health beliefs and attitudes toward death play an important role in their decision-making [10].

Clearly, the decision to initiate warfarin therapy is a complex interaction of many variables, involving patient, provider, and system factors. Most studies examining anticoagulation practices in primary care have used surveys or other forms of quantitative methods; however, given the inherent complexity of this subject matter, it is likely that qualitative research methods could provide significant additional insight. A comprehensive literature search failed to find a qualitative investigation of patient perspectives and experiences taking warfarin. The aim of this study, therefore, was to employ qualitative methods to examine the experience and perspective of individuals on long-term warfarin therapy for atrial fibrillation as a means to assess the extent to which the physician-identified barriers reported by Bungard [6] are in concordance with the unique views of patients.

## Methods

### Participants and setting

This research was undertaken in a family practice clinic situated in a large, tertiary care teaching hospital. The clinic employs 12 physician full-time equivalents working in three teams; each four-physician team is supported by two registered nurses. The practice, which serves a medium-high socioeconomic status population, has a large proportion of elderly patients and the prevalence of AF is higher than reported elsewhere [1]. The prevalence of atrial fibrillation in our population as a whole is 3.9 percent. When considering different age groups, the prevalence rises as high as 18.2 percent and 18.5 percent for patients aged 80–89 and 90–99 years, respectively. A recent chart audit study found that the majority of eligible AF patients in the clinic (78%) are being treated with warfarin for stroke prevention [11]. Comprehensive anticoagulation services are provided and the clinic offers access to physicians on-call 24 hours a day. The nurses maintain an historical record of International Normalized Ratio (INR) results and warfarin dosage changes for each individual patient and, in consultation with the physicians, inform patients of prescribed warfarin dosage changes in a timely fashion, usually on the same day as the INR reading.

Potential participants were identified by the clinic nursing staff. The inclusion criteria stated that the patient must currently be on warfarin therapy and have been so for a minimum period of six consecutive months. Patients were excluded from the study if a significant co-morbidity prevented their participation, if they were unable to converse in English, or if they were unwilling/unable to provide informed consent. From a pool of approximately 60 eligible candidates, the nurses purposively sampled in order to achieve an even gender split as well as an equivalent number of patients who were both normally in-range and out-of-range on their INR tests. A total of 24 patients were invited to participate in the study. Three patients declined to take part, two of whom expected to be unavailable during the interview phase, whereas the third declined due to lack of interest. All participants signed informed consent forms in advance of their participation in the study, which was approved by the Research Ethics Board of the host institution. A demographic profile of the sample is presented in Table 1. The mean age of participants was 74 years; there were 12 males, 9 females. The majority were both married (86%) and retired (86%). The mean length of time participants had been on warfarin therapy was 4.6 years (range = 1 year to 10 years).

**Table 1:** Demographic profile of participants

**Code**	**Age (years)**	**Sex**	**Marital Status**	**Employment Status**	**Years on Warfarin**	**INR in Range?**
P1	67	F	Widowed	Retired	5	Yes
P2	73	M	Married	Retired	10	Yes
P3	84	M	Widowed	Retired	5	Yes
P4	83	F	Married	Retired	4	No
P5	81	F	Married	Retired	1	Yes
P6	76	F	Married	Retired	3	Yes
P7	75	M	Divorced	Retired	2	No
P8	60	M	Married	Working	3	No
P9	79	F	Married	Retired	5	No
P10	67	F	Married	Retired	2	No
P11	76	M	Married	Retired	5	No
P12	80	M	Married	Retired	2	No
P13	53	M	Married	Working	8	No
P14	71	M	Married	Retired	3	Yes
P15	69	M	Married	Retired	4	Yes
P16	77	F	Married	Retired	5	Yes
P17	78	M	Married	Retired	1	No
P18	80	M	Married	Retired	10	Yes
P19	71	M	Married	Working	7	No
P20	71	F	Married	Retired	6	No
P21	82	F	Married	Retired	5	Yes

### Data collection and analysis

We utilized a semi-structured interview guide that was developed on the basis of salient issues identified in the scientific literature, specifically the various barriers to the prescription of warfarin for atrial fibrillation as reported by Bungard et al [6]. Interviewees were asked to share their experiences with warfarin in relation to four specific content areas or themes: decision-making, knowledge and education, impact on daily life, and patient satisfaction. Throughout the course of the interview, participants were provided several opportunities to raise issues or to describe experiences that had not been specifically addressed. Standard demographic information was also collected. Three of the authors (GCD, JM, and BT) shared the task of conducting the interviews. The protocol for assignment of each individual participant to an interviewing author ensured that there had been no previous clinical contact between the two parties; moreover, interviewers were blind to interviewees' INR status. The interviews lasted an average of 45 minutes and took place either in the clinic (n = 13) or in participants' homes (n = 8), in accordance with each participant's preference. Data collection ceased when, in the consensus of the research team, saturation had been reached; that is, no new ideas or perspectives were emerging. All interviews were audiotaped and transcribed verbatim.

Given the pre-determined nature of the themes, as described above, we employed a content analysis approach to the analysis of the interview data. Whereas grounded theory is used to develop data-induced themes or hypotheses, content analysis is the better-suited approach in those instances where the codes, categories, or themes of interest to the investigators have been previously discovered and described [12], as is the case in the present study.

Precise criteria were developed for each of the four pre-determined themes in the codebook, namely decision-making, knowledge/education, impact on daily life, and patient satisfaction. The 21 transcripts were then coded according to these criteria. Each transcript was coded by at least two members of the research team using a standardized coding form. Tests for inter-coder reliability indicated a high level of agreement among the coders; instances of disagreement were resolved through a process of discussion and negotiation that included both the fourth author and the principal investigator (CST & REGU). This process yielded a unit-by-variable matrix that allowed for substantive analysis of the data. In order to strengthen the validity of the findings, the analytic processes of coding and interpretation were reviewed by an independent external reader (DG).

## Results

### Decision-making

The great majority of participants reported that the decision to initiate warfarin therapy had been made by "the doctor" – a term that was used to refer not only to family physicians and general practitioners, but also to specialists and attending physicians in urgent care settings. Typically, there was little or no patient involvement in the decision-making process (Table 2A). In most cases, this unilateral decision-making appeared to be related to the high level of trust that patients place in the medical expertise of physicians; indeed, the phrase "doctor knows best" was commonly-used in these accounts (Table 2B). For a smaller number of participants, the specific circumstances surrounding the initial decision to commence therapy served to preclude any degree of significant involvement on their part (Table 2C).

**Table 2:** Quotations: Decision-making

***A. Minimal patient involvement***
My decision [to take warfarin]? It was the doctor's decision. **(P5)** I had nothing to say [regarding decision to initiate warfarin]. If the doctor tells me something, I do it. **(P17)** Q: What influenced your decision to take the drug? A: Well, because I was told to. Q: The main reason is that it was the physician's recommendation? A: Yes. **(P20)** I don't recall him [the physician] saying anything much. He said a lot of things when he examined me first, and he put me up in the ward overnight, then he started with the medications. That's all there was to it...Not really, no [no much discussion on reasons to start warfarin]. He just said that, "*This is what medication we're going to put you on for the myopathy." *That was it. **(P11)**
***B. Trust of physicians***
I just figured the doctor knows best... **(P1)** A: No [trouble to decide to take warfarin], because I knew nothing about it. My doctor, as far as I know, is very competent so... Q: So you are taking it basically because the doctor told you to? A: That's right. **(P6)** I'm at this hospital and it's got a very good reputation... Doctor knows best, I guess. They know exactly what you have to do for it, and they did it. **(P14)** I can recall that I had no objection. I said, "*You are the experts, you are the doctors. If I get any help, I mostly will appreciate it*.".... I don't think I would trust myself that much [to make the right decision]. **(P15)**
***C. Constraining effect of circumstances***
When I went into the [clinic] to see my doctor, they admitted me to the cardiac emergency, and they kept me there all day ... I was in for just about a week. ... and when I was discharged the doctors explained that they were putting me on to certain medications, and Coumadin was one of them. **(P10)** I had congestive heart failure, that's what I was in hospital for. I don't know what I was on when I was in hospital, but when I came out I had a whole slew of medications and Coumadin was one of them. **(P5)** I had lymphoma, and then I had a bone marrow transplant for lymphoma, and I had my spleen taken out, and I started getting deep vein thrombosis. Then I had a pulmonary embolism at one point and they started me on it [warfarin] then... I had just about every complication in the book, and this [thrombosis] was one of them. I think it was around that time, or within a year after the thromboses started, they gave me warfarin. **(P13)** The surgeon said I had to take it, basically. I don't like taking pills, so when I went in to get my one valve replaced, they gave me – they persuaded me – and I agreed to trying out something new that had just been approved. The reason for doing that was that I would not have to take Coumadin... Unfortunately, however, one of my other valves blew when I was in there [during surgery], so I got two for the price of one, and then there was no question I had to go on a blood thinner. **(P19)**

### Knowledge and education

The level of knowledge and understanding of the benefits and risks associated with warfarin therapy tended to vary with age. Elderly patients (aged 75+) demonstrated poorer knowledge than their younger counterparts; indeed, the knowledge level among older participants appeared to be quite superficial and scattered (Table 3A). Whereas elderly patients could not explain with any degree of exactitude the rationale for taking warfarin and the associated risks, for a subset of participants, most of whom were less than 75 years old, the knowledge level was higher and, for a small number, considerably higher (Table 3B). Overall, less than half of our sample was able to name one specific benefit, risk, and lifestyle change/concern associated with warfarin therapy.

**Table 3:** Quotations: Knowledge and education

***A. Superficial level of knowledge***
I don't really know what these different pills do for me. **(P3)** I'm assuming these people know what they're doing. They're not doing this for nothing. They must have good reasons, and they tell me, "*Hang on there, you're doing all right. Keep it up.*" So I do. I don't question them. Very little, if any. I probably wouldn't know what they were talking about if they started to explain it all, and what's the point of that? **(P12)** Q: What do you think Coumadin is doing for you and your health? A: It makes the blood sticky, I believe, or thins it. I really don't know. Q: Do you know why they added the Coumadin? A: No idea. Q: It doesn't much matter to you? A: It doesn't matter to me. Q: Everyone's different. Some people like to know all the details. A: Oh, I couldn't care less, just as long as it keeps me alive. **(P17)** I hope it's [warfarin] keeping everything under control... Well, the stroke that I had, I don't feel sick, I don't have any pain, or anything. **(P21)**
***B. Superior knowledge of risks and benefits***
A 72-year-old male has a 30 percent chance of having a stroke regardless, but if I didn't take the Coumadin, it would be a 70 percent chance of having one. So I'm taking medication to avoid the stroke. **(P14)** Nobody really explained to me in full what Coumadin is all about, but I did some reading about it. I know it's a blood thinner, an anti-coagulant... helps with the atrial fibrillation that I have, because apparently blood stays longer than it should in the atrium, and if it thickens it can go to your brain and you can have a stroke. **(P8)**
***C. Patient education***
Q: When you started on the Coumadin, did you receive any education about the medication? A: Just that the doctor said to me that it's not 100 %, like anything else, but there's less chance [of stroke]. **(P16)** I said to the nurses once, "*Supposing I stop taking it." *They said, "*Oh, I wouldn't advise it, you know, because within a month, you'd have the most severe stroke, or it would kill you, one or the other." *That scared me.**(P3)** Q: Do you remember if you received any educational material about Coumadin? A: No. Q: Or any talk about how it works, the benefits? A: Not that I can remember. I cannot recall that, no. Q: Any pamphlets, any coloured paper, anything? A: No. Q: You don't recall? A: If I did have, I read it, then I dismissed it... I have an appetite, I can eat and drink, I can sleep, and I still can work. Anything else, to me, is not quite important. It's probably wrong. I should read them and pay more attention. **(P15)** Then he [physician] said, "*If you go off the Coumadin for your operation, you could get a stroke. You've got a choice: you can either go off the Coumadin or you can stay on it and bleed to death." *Not to death, he didn't say that. You'd bleed. The other way, you could have a stroke. That's all he said. So I presume that it could happen. **(P9)** Q: Is there any sort of educational material that you would like to see on warfarin? A: No. I've got a pile of books to read now, and as soon as I start to read, I fall asleep. The pamphlets would fare worse than the books. I don't think that would help. **(P12)** Q: Did you have many questions about it at the time [when warfarin was initiated]? A: No. You see, the darn trouble was that my wife would be sitting there, and I'd say, "*She knows what it's all about; tell her." *... When you have a sit-in nurse, you know, I don't worry about that stuff [getting education on the therapy]. **(P3)**

According to participants' accounts, educational efforts aimed at informing patients about warfarin were minimal and insufficient (Table 3C). Those who were able to recall some form of education typically referred to a "booklet" or "sheet" supplied by either the clinic or a pharmacy. In several cases, spouses were more knowledgeable than patients and appeared to play an important role in monitoring the regime. A number of participants lauded the availability of clinic staff to answer questions; however, two others reported the use of "scare tactics" by health care professionals with regard to the need to take warfarin.

### Impact of warfarin regime

While there is tremendous range in the perceived impact of warfarin therapy on the lives of these patients, the vast majority reported that they have not experienced complications (e.g., hemorrhage, drug interactions). Typically, the decision to start taking warfarin did not precipitate significant changes in their day-to-day lives; many participants reported experiencing only minor inconveniences (Table 4A). For these individuals, warfarin is just another pill to be taken everyday; many reported the use of some reminder strategy, such as calendars, dosettes (pill boxes), or taking the pill right before some regular activity, in order to avoid missing a dose. On the other hand, a sizeable proportion (25%) of interviewees reported that adhering to the warfarin regime does impact upon their day-to-day lives. From the perspective of these patients, who were more likely to have multiple co-morbid illnesses and/or were taking multiple medications, the warfarin regime presents a considerable struggle to be managed, particularly when dosages needed to be adjusted. Regular visits to the clinic, restrictions on diet and alcohol intake, and anxiety regarding bleeding and potential drug interactions counted among the most commonly cited impacts (Table 4B). Only a small number of participants reported experiencing significant complications related to the warfarin regime, with one case involving repeated gastro-intestinal bleeding. These patients demonstrated a very high level of commitment towards their warfarin management and have placed the ritual of taking the medication at the centre of their daily routine (Table 4C).

**Table 4:** Quotations: Impact of warfarin regime

***A. Minor impact***
Coumadin, it's just a matter of taking the pills each day and coming for a blood test and adjusting the dose. That's all. No other impact, as far as I'm concerned. **(P13)** I had the test [INR] done before I went to Europe. I arranged it so that two days before we flew to Europe, I had it tested, and it was 2.3, I think... The nurse said: "*You might keep it this same way, and enjoy.*" And as soon as I got back, I came in and had it checked. **(P15)** It's inconvenient having to come in every week, but on the other hand, I understand. **(P20)**
***B. Moderate impact***
I will only drink one glass of wine a day. I like a glass of wine. They say just go easy on the single malt, and stuff like that...There wasn't any special [instructions regarding diet]. We like good food, and we eat a good, balanced diet. I like seafood, and I love fish, and I like the odd steak. I try to stay off butter. I'm taking Becel^® ^just now, which I don't really like, but I try to stay off the butter and cooking with all the white sauce, and butter sauce, and stuff like that. **(P10)** I come here every 4 or 5 weeks to have the bloodwork done for Coumadin. Whenever I have this done, the nurse calls me that afternoon and says, "S*tay on with the same milligrams" *or "*Change to that and that." *But I feel fine [with this routine]... The only disadvantage of the Coumadin is the bruising and bleeding on just the slightest touch... but if that's the worst that happens, I'm not worrying about it. **(P11)** I'm extremely careful with my alcohol intake, although as I said before, I'm not an everyday drinker. Other than that, the only other thing is I started noticing I have experienced some hair loss. **(P8)**
***C. Major impact***
Three years ago, I had two very serious stomach bleeds and I do not know, to this day, whether I should attribute it to Coumadin. Once I spent a couple of days in intensive care and then three months later, again a couple more days, this time in critical care. **(P11)** It isn't worth it to risk a stroke by going off the Coumadin to have the hernia fixed. So Coumadin has played a major part in my life, because this hernia is a daily fact I have to live with... The fact that I'm taking Coumadin means that if I want to be operated on, I have to be careful... They [the Hernia Clinic] don't take guys like me that require a bit of time and skill and more facilities than they have. But what do guys like me do? **(P14)** The thing is, the last 558 times I've taken it, which is right here [referring to the records he keeps], I went through this last night, and I found only three miscues the entire time I've been taking it. That works out to 0.005 percent. It's a very, very low percentage of goofing taking it. I've never forgotten. For at least two of these incidents, I took it at 10:00 or 10:45, instead of at 6:00. That's four hours late. I consider that a goof. Another time I took it in the morning instead of at night, which is a goof. **(P14)**

### Patient satisfaction

The vast majority of interviewees reported a high level of satisfaction with the care they receive from the nurses and physicians in the clinic (Table 5A). Most participants were also satisfied with the warfarin regime itself (Table 5B). Only a very small number of participants expressed significant dissatisfaction. The sources of dissatisfaction, which tended to be highly localized to specific concerns, included the cost and inconvenience of attending the clinic for regular INR tests, a lack of information provided to patients, and insufficient awareness of patient history on the part of clinic staff (Table 5C).

**Table 5:** Quotations: Patient satisfaction

***A. Satisfaction with clinic staff***
Oh, I am more than pleased. I'm absolutely more than pleased. I think they're wonderful. The nurses are wonderful – you know, taking my blood, and phoning me, and giving me instructions. **(P1)** I think they've been 100 percent. From my cardiologist to the family physician and to the pharmacists, because they're just amazing. **(P10)** My doctor thought my blood was, I guess, too thick. They did an INR and recommended the Coumadin, which I didn't want to get on, because I knew it was warfarin and you associate that with rat poisoning. Anyway, she took the time and very patiently explained what the purpose of it was, and highly recommended it. I really like her. I like everybody there. They're very caring, supportive people. They're just dear. **(P4)**
***B. Satisfaction with warfarin regime***
This is a pill that keeps your blood thin, and you have to check it out [INR level]. I just do as I'm told and I'm thrilled that they keep me at 2-point-something....I go every week, or every other week, or once a month, depending on my stability. It doesn't bother me going. **(P1)** It's worked out well [the regime]. I know it has to be done, and I'm lucky in the fact that it has regulated it. It has totally regulated itself – I'm taking 4 mg a day now. I'm only coming in once a month. **(P18)** Oh, yes [happy with warfarin regime]. They let me know what the status is each week, whether it's going to level out, and whether I'm going to be able to stay on the same dosage and then stop going up there every week. I started going every week, and now it's been levelled to every two weeks. **(P7)** I really couldn't say anything bad about it [warfarin regime]. Apparently I've been outstanding in how steady I would go with it, and I've been going – normally, for years, I've been going once a month, pretty well. A couple of times it would go up and I'd come back in two weeks, or something. **(P2)**
***C. Sources of dissatisfaction***
Nobody tells me anything. That's one of my problems with this whole bloody business. Nobody tells me how I'm doing. All I know is that I'm supposed to be between 2 and 3 [INR levels]. **(P7)** I just more or less come when I'm ordered to. From home it's almost an hour on the bus each way, and the parking around here, the cost is wild. They must be financing the place with the parking. No, I would prefer not to come at all. I would prefer to forget the whole deal, but that doesn't seem to be in the offing at the moment. **(P12)** I haven't been coming here that long, about two years I think... but I wouldn't say they're fully aware of my history and really understand the depth of it... Considering my history, I think they should know more. They certainly don't have my files. **(P13)**

## Discussion

The findings of this study provide significant and original insight into the perception and experience of patients taking warfarin. The data indicate that patients tend to have minimal input into the decision to initiate warfarin therapy; many have only a superficial level of understanding of the risks and benefits of warfarin; and the majority retain little from the education they received regarding warfarin therapy. This outcome is balanced, however, by the finding that for these patients there was both a high level of satisfaction with the care provided in the family practice setting and a low level of impact on their day-to-day lives.

The principal strength of this study is the insight into the lived experience of warfarin therapy as gleaned from the unique perspective of family practice patients currently taking warfarin. We view this as a significant and novel contribution to the literature as we could find no other such study. It is important to note, however, that our sample was drawn from the patient population of an academic primary care practice that is both well-educated and of medium-high socioeconomic status. The applicability of these findings in other patient populations may therefore be limited.

Interviewees revealed clear detachment from participation in the decision-making process around initiating warfarin therapy. In some cases, this detachment appeared to stem from the particular circumstances at the time; for instance, if the patient was involved in a medical emergency or was admitted to hospital and had several medications initiated. For others, there was a general belief or understanding that warfarin is a medication without which the patient faced imminent risk of death – it is therefore not a matter to be discussed or negotiated. This finding may be a function of the age of our participants. As a group, elderly patients tend to prefer a directed rather than shared consultation. Prior research indicates that seniors are more likely to be accepting of medical advice without much questioning, rather than assuming a more active role in the decision-making process [13]. This attitude was reflected in participants' comments that "doctor know best" and "it's the doctor's decision."

Patient knowledge of risks, benefits, and issues related to diet and alcohol intake was low, although younger patients demonstrated greater levels of understanding than did those over age 75. This finding is consistent with previous investigations. Lip and colleagues have detected lower levels of knowledge among elderly patients; moreover, longer duration of anticoagulation does not appear to ameliorate patient understanding significantly [14,15]. For the most part, we found that retention of instructions pertaining to the warfarin regime was poor. Many participants reported that they simply follow the nurses' directions and have little interest in learning anything more. The limited knowledge and seemingly low level of interest to learn more could be attributable to the great deal of trust invested in the expertise of the clinic staff as discussed above. The fact that several spouses exhibited greater understanding of the risks and benefits associated with warfarin therapy has implications for educational interventions recognizing the importance of the spousal or care giver role in the monitoring of therapy.

With regard to the impact of warfarin therapy on daily life, our results indicate that warfarin is for the most part well tolerated and does not pose heavy additional burdens or lifestyle changes. The majority of participants were already taking several medications and visiting more than one doctor on a regular basis. This sample also had a low incidence of complications and previous studies have shown that the potential for complication does not on its own result in a significant impact [16,17]. That is, the mere possibility of an adverse side-effect does not bring about substantial anxiety unless the complication is actually experienced. These results do not, however, capture the experiences of individuals who have tried and ceased taking warfarin for whatever reasons. This population represents a high priority for future study. Additional support may be required for individuals with multiple medical problems. Continuity of care and a strong relationship and identification with the primary care team that provides anticoagulation services can overcome potential miscommunication and misunderstanding regarding side effects and drug interaction.

The importance of the association between patient satisfaction and adherence has been established in prior research on anticoagulation. In a case-control study, Arnsten and colleagues [18] found that, among patients with a regular physician, the non-adherent cases were those who expressed dissatisfaction. In our sample, the level of patient satisfaction was high, both with the clinic staff and the warfarin regime itself. Based on participants' testimonies, the coordination and continuity of care by a trustworthy team of doctors and nurses were key contributing factors to the high satisfaction ratings. In an evaluation of a telephone-based anticoagulation service, Waterman found that patient satisfaction with warfarin management was associated with the timeliness of receiving blood test results from the service provider [19]. The high level of patient satisfaction observed in the present study may also be due in part to the low rate of complications (e.g., hemorrhage, drug interactions), which may serve to reinforce patients' trust both in the therapy and in the health care team.

Increasingly, theoretical models of the physician-patient encounter advocate the inclusion of patients in the decision-making process [20]. Of course, shared decision-making presupposes an understanding of the benefits and risks on the part of patients. With regard to warfarin therapy, patient preferences would be expected to vary according to expected benefits or awareness of risks of suffering a stroke. Man-Son-Hing et al have demonstrated that the minimal clinically important difference of warfarin therapy is often considerably smaller for patients than that identified by clinicians [21]. Protheroe and colleagues, in an observational study of patient-based decision analysis, noted marked disagreement between patient preferences and guideline recommendations [22]. A patient decision aid was shown to improve knowledge and understanding of the risks and benefits of warfarin for patients with atrial fibrillation, and aided in therapeutic choice [23]. Given the low level of patient knowledge observed in the present study and elsewhere, the vision of shared decision-making [24] remains an as yet unachieved, but laudable goal; indeed, the present results highlight the challenges of shared decision-making and increased autonomy in patients with complex chronic diseases.

## Conclusions

In summary, the results of this study suggest that patients tend to have limited input into the decision to initiate warfarin therapy. Moreover, a majority appear to lack a comprehensive understanding of the risks and benefits associated with treatment. These findings, however, were balanced by the minimal impact of warfarin on daily life and the high level of patient satisfaction. Further research is required to assess whether these findings are similar in other patient groups, with different demographic and socioeconomic characteristics, including multi-cultural communities [14]. Investigation of physician views of the underutilization of warfarin therapy would allow for a comparison of the patient and provider perspectives. Clearly, there is a pressing need for innovative methods of continuing patient education in order to communicate the risks and benefits of warfarin therapy in a friendly, non-threatening manner. Also, these results highlight the tremendous influence that care providers exert on the decision-making of patients. The development of decision aids for anticoagulation may help patients make more informed decisions [23,25], but only if care providers know of their existence and take the time to use them, assuming that such tools are feasible in a busy clinical setting.

## Competing interests

None declared.

## Author's contributions

REGU conceived and initiated the study and will act as guarantor. GCD, JM, and BT conducted the interviews. CST co-ordinated the data analysis process. All authors participated in the analysis of the data and the writing of successive drafts of the manuscript and all have read and approved the final draft.

## Pre-publication history

The pre-publication history for this paper can be accessed here:


